# Exposed and Sequestered Antigens in Testes and Their Protection by Regulatory T Cell-Dependent Systemic Tolerance

**DOI:** 10.3389/fimmu.2022.809247

**Published:** 2022-05-26

**Authors:** Jessica Harakal, Hui Qiao, Karen Wheeler, Claudia Rival, Alberta G. A. Paul, Daniel M. Hardy, C. Yan Cheng, Erwin Goldberg, Kenneth S. K. Tung

**Affiliations:** ^1^ Department of Pathology, University of Virginia, Charlottesville, VA, United States; ^2^ Department of Microbiology, University of Virginia, Charlottesville, VA, United States; ^3^ Bierne B. Carter Center for Immunology Research, University of Virginia, Charlottesville, VA, United States; ^4^ Cell Biology and Biochemistry Department, Texas Tech University Health Science Center (HSC), Lubbock, TX, United States; ^5^ Center for Biomedical Research, Population Council, New York, NY, United States; ^6^ Molecular Biochemistry Department, Northwestern University, Evanstan, IL, United States

**Keywords:** testis autoantigens and autoantibodies, exposed and sequestered testis autoantigens, the residual bodies and cytoplasmic droplets, experimental and human autoimmune orchitis, post-vasectomy tolerance versus orchitis, Foxp3+ regulatory T cells and systemic tolerance

## Abstract

Continuous exposure of tissue antigen (Ag) to the autoantigen-specific regulatory T cells (Treg) is required to maintain Treg-dependent systemic tolerance. Thus, testis autoantigens, previously considered as sequestered, may not be protected by systemic tolerance. We now document that the complete testis antigen sequestration is not valid. The haploid sperm Ag lactate dehydrogenase 3 (LDH3) is continuously exposed and not sequestered. It enters the residual body (RB) to egress from the seminiferous tubules and interact with circulating antibody (Ab). Some LDH3 also remains inside the sperm cytoplasmic droplets (CD). Treg-depletion in the DEREG mice that express diphtheria toxin receptor on the Foxp3 promoter results in spontaneous experimental autoimmune orchitis (EAO) and Ab to LDH3. Unlike the wild-type male mice, mice deficient in LDH3 (wild-type female or LDH3 *NULL* males) respond vigorously to LDH3 immunization. However, partial Treg depletion elevated the wild-type male LDH3 responses to the level of normal females. In contrast to LDH3, zonadhesin (ZAN) in the sperm acrosome displays properties of a sequestered Ag. However, when ZAN and other sperm Ag are exposed by vasectomy, they rapidly induce testis Ag-specific tolerance, which is terminated by partial Treg-depletion, leading to bilateral EAO and ZAN Ab response. We conclude that some testis/sperm Ag are normally exposed because of the unique testicular anatomy and physiology. The exposed Ag: 1) maintain normal Treg-dependent systemic tolerance, and 2) are pathogenic and serve as target Ag to initiate EAO. Unexpectedly, the sequestered Ags, normally non-tolerogenic, can orchestrate *de novo* Treg-dependent, systemic tolerance when exposed in vasectomy.

## 1 Introduction

Inbred mice thymectomized between day 1 and day 5 (d3tx) after birth spontaneously develop autoimmune disease in the ovary, prostate, stomach, testis and/or lacrimal gland (1). All the diseases are preventable by the early implantation of lymphoid organs from normal syngeneic donors (1–3). This seminal observation led to discovery of the CD4+ Foxp3+ CD25+ regulatory T cells (Treg) crucial for organ-specific autoimmune disease prevention (4–6). Peripheral Tregs may develop “naturally” in the thymus (nTregs), or may have converted from Foxp3-negative “conventional” CD4+ T cells in the periphery (pTregs) (7). The CD4+ nTregs develop in response to tissue auto-peptides expressed in the thymic medulla – a process controlled by unique Ag presenting dendritic cells (8), the transcription regulator *AIRE* (9), the transcription factor *Fezf2* (10), and the chromatin remodeler Chd4 (11). Relevant to this review are 1) the idiopathic male infertility that occurs in autoimmune polyendocrinopathy syndrome 1 with or without *AIRE* mutation (12, 13), 2) the orchitis detectable in the *Fezf2* NULL mice (10), 3) the Tregs that develop in the periphery in response to self or foreign peptides presented by tolerogenic Ag presenting cells in appropriate cytokine environment (14–16), and 4) autoimmune sequel of human vasectomy.

Tregs control T effector cell responses in the regional lymph node (LN) of the target organ and prevent autoimmune disease (17). There is enrichment of Treg function in the normal regional LN for prevention of disease in the target organ. Whereas 0.5 million Tregs pooled from all LNs of normal mice are required to adoptively suppress autoimmune ovarian disease in d3tx mice, only 30,000 Tregs from the ovary-draining LN are sufficient (17). This finding applies equally to the Tregs in LN draining the prostate and lacrimal glands (18). The enrichment of disease-specific Treg in regional LN requires continuous exposure to relevant Ag from the target organ. For instance, ablation of prostate Ag by neonatal orchiectomy deprives prostate-specific Treg enrichment in the prostate-draining LN without affecting Treg enrichment in LN draining the lacrimal gland. Indeed, Treg enrichment in the prostate-draining LN can be quickly restored in the orchiectomized mice when prostate Ag are re-expressed upon testosterone treatment (18–21). These findings, initially conducted using polyclonal Tregs, have been confirmed and extended by studies using Ag-specific Tregs (21, 22). Therefore, Ag accessibility, controlled at the level of the target organs can influence systemic tolerance initiated in the thymus (23–25). In this context, the sperm Ag in the normal testis has long been considered unique in that they are all sequestered from the immune system and are beyond regulation by systemic tolerance.

The Ag relevant to experimental autoimmune orchitis (EAO) are expressed in post-meiotic germ cells inside the seminiferous tubules (SFT). They are separated from the interstitial space by the blood-testis barrier (BTB) provided by the Sertoli cells with their basal and apical tight junctions (26). Rodent sperm Ag appear at about 2 weeks after birth (27). Thereafter, a large quantity of numerous novel testis-specific antigens are synthesized and expressed in the meiotic germ cells (28). Murine spermatogenesis occurs in 12 stages along the length of an adult seminiferous tubule, each defined by unique composition and arrangement of the germ cells (29). However, the first wave of spermatogenesis initiated at puberty is synchronous for all tubules (30). After they differentiated from the haploid spermatids, sperm released into the seminiferous tubular lumen would traverse the straight tubules, the rete testis, the ductus efferentes, and enter the caput epididymis. Further transit through the epididymis body and tail complete sperm maturity (28). This paradigm of testis biology has led to the concept that sperm Ag of late ontogeny are sequestered in a completely closed epithelial environment; they do not communicate with the immune system including with Tregs, and are unable to maintain “neonatal” tolerance (28, 31–33). Accordingly, local Ag independent mechanisms (or local immune privilege), operating in the testis and epididymal interstitial spaces, are essential for immune protection of testicular Ag. The idea of Ag sequestration was also a rationale behind the application of highly immunogenic cancer/testis Ag (CTA) as cancer vaccine candidates (34). However, sperm Ag sequestration, the lack of systemic tolerance, and the natural hyper-immunogenicity of CTA are not evidence-based.

Several studies have argued against the complete sequestration of sperm Ag. First, mice immunized with testis homogenate in incomplete Freund’s adjuvant do not develop EAO but they produce Ab that react with the diploid preleptotene spermatocytes located outside the BTB, indicating that germ cells outside the BTB are immunogenic (23, 35). Second, d3tx mice develop autoimmune orchitis and epididymitis, and the disease is prevented by infusion of T cells from normal syngeneic donors. This suggests the existence of testis-specific Treg (36). Third, the preferential accumulation of inflammatory cells at the straight tubule in adoptive EAO transfer (37), is likely due to reduced mechanical barrier in this location (38). Nonetheless, a long-standing question remained. How do testis-specific T cells receive their tolerogenic Ag signal, and how do effector T cells and Ab engage their target sperm Ag in the testis to initiate EAO if the Ag are completely sequestered?

The first definitive evidence against complete sperm Ag sequestration was obtained in a study that documented continuous sperm Ag egress from normal SFT and the protection of the exposed Ag by Treg-dependent systemic tolerance (25). Herein, we review the published experiments, including the mechanism of Ag egress and systemic tolerance controlled by the Tregs. We also document that some other sperm Ag, which lack the properties of the exposed sperm Ag, are indeed sequestered. We first encountered the sequestered sperm Ag in 2011 when we investigated mice with unilateral vasectomy (Uni-Vx) (39). We were puzzled by the absence of the anticipated post-vasectomy robust Ab response to sperm. Instead, we found rapid induction Ag-specific Tregs against ZAN, an acrosomal sperm Ag subsequently confirmed to be sequestered (25, 39, 40).

## 2 Findings

We present our results in two parts. Part 1 will summarize our published studies on the responses to three individual sperm Ag in normal mice (25). Part 2 will describe the findings from our earlier research on the Uni-Vx mice, which, in retrospect, is highly pertinent to our findings in Part 1 (39, 40).

### 2.1 Part 1: Accessibility and Immunological Studies on Meiotic Germ Cell Antigens Located in Seminiferous Tubules of Normal Mice

#### 2.1.1 Detection of LDH3 and Transgenic Ovalbumin Egress From the SFT to Interstitial Space

Lactate dehydrogenase 3 (LDH3) is a cytoplasmic Ag of meiotic germ cells located inside normal ST. LDH3 first appears at postnatal week 2. To detect LDH3 egress from the SFT, we injected rabbit Ab against mouse LDH3 intravenously into normal 8 week-old male mice (27). Twelve hours later, we detected immune complexes as granular deposits of rabbit IgG in mice injected with LDH3 Ab. The immune complexes were localized outside the BTB and surrounded 20% of the SFT (25). To support the LDH3 specificity of the immune complexes, we did not detect them in the *Ldh3* null mice injected with LDH3 Ab or in wildtype (WT) mice injected with Ab against ovalbumin (OVA). In addition, immune complexes were not detected beyond the testis, including the caput, corpus, and cauda epididymis.

We also investigated the sperm Ag zonadhesin (ZAN), located in the inner acrosomal membrane (41). In contrast to LDH3, we did not detect evidence of egress of ZAN, a sperm acrosomal Ag. Therefore, both LDH3 and ZAN exist in the SFT behind the BTB, but only LDH3 egresses from the SFT. While the Sertoli cells and the BTB may prevent the entrance of macromolecules into the SFT lumen (42), they do not inhibit the egress of all sperm Ag into the interstitial space.

#### 2.1.2 LDH3 and Transgenic Ovalbumin Egress From the Seminiferous Tubule as Contents of the Residual Body

Following LDH3 Ab injection, immune complex formation is dependent on the age of the mice; they are detected in 5 week-old mice but not in mice at the ages of 3, 4, or 4.5 weeks. The onset at 5 weeks coincides with the onset of spermiation, a process wherein elongated haploid spermatids transform into sperm in the first wave of synchronous spermatogenesis (30). At spermiation, the redundant cytoplasm and plasma membrane from the late spermatid are packaged into: 1) a large residual body (RB) retained in the SFT, and 2) a smaller cytoplasmic droplet (CD) that remains attached to the sperm until they leave the caput epididymis (25, 43, 44). To investigate the hypothesis that LDH3 is exported as the content of the RB, we utilized mice expressing transgenic OVA on the protamine 1 promoter (the OVA-Hi), at 10 μg of extractable OVA per gram of testis, as a surrogate sperm-specific cytoplasmic Ag in elongated spermatid and sperm (25). We also utilized the Scleraxis-GFP mice with Sertoli cell-specific fluorescence (25) as well as Ab to the testis isoform of angiotensin-converting enzyme as a RB marker (45).

We detected LDH3 in the RB of normal WT mouse testis by indirect immunofluorescence. Intense staining of OVA was also noted in the RB of the OVA-Hi mice that co-localizes with LDH3. Detection was maximal at stage IX of the spermatogenic cycle (25). Some RB, engulfed by Sertoli cells, appears as dual-fluorescence labeled star-shaped objects. However, some RB retains their normally round shape and are distributed from the apex to the base of the SFT epithelium. Importantly, these RB stain positively for OVA and LDH3 and have no Scleraxis-GFP co-stain (not shown). Thus, they are located outside the Sertoli cell cytoplasm. In addition, strong OVA staining appears inside the basal cytoplasm of the Sertoli cells, as droplets in the adjacent interstitial space in OVA-Hi mice, and inside interstitial macrophages. To show OVA egress from the SFT, we intravenously injected OVA-Hi mice with OVA Ab. This led to immune complex formation on the surface of SFT outside the BTB, some inside the interstitial F4/80+ macrophages.

Therefore, not all RB are engulfed and destroyed by the Sertoli cells. Some RB located outside the Sertoli cells remain intact until they reach the base of the Sertoli cells. At that location, spermatid Ag appears to be transported into the interstitial space *via* the basal Sertoli cell cytoplasm. These findings are consistent with an electronic microscopic study of labeled rat RB by Xiao et al. (46). After sperm are released at stage IX, the RB were described to move rapidly to the edge of the SFT, change into lipid inclusions and lipid particles, and were discharged into interstitial space. However, the definitive movement of sperm Ag from Sertoli cell to the interstitial space requires more detailed investigation.

The differential sequestration status between LDH3 and ZAN is confirmed recently based on proteomic and mass spectroscopic analyses of the testis interstitial fluids and serum proteins (47). The authors proposed two hypothetical mechanisms for meiotic Ag egress from the ST. One is *via* the RB at stage IX of the spermatogenic cycle (25) and the second to occur earlier, at spermatogenic cycle stages VI and VII prior to RB formation.

#### 2.1.3 Immunological Evidence for: A) Exposed Status of LDH3 and Transgenic OVA, B) Tregs as Their Tolerance Mechanism, and C) Sequestered Status of ZAN

First, we detected differences in the immunogenicity of LDH3 and ZAN between Ag-positive mice and Ag-negative mice (23, 25). WT male C57BL/6 mice immunized with testis homogenate in adjuvant do not produce Ab to LDH3. In contrast, robust LDH3 Ab responses are elicited in both LDH3-deficient *Ldh3* null male C57BL/6 mice and female WT C57BL/6 mice. Importantly, the treatment of the LDH3-immunized WT male mice with CD25 Ab (PC61), which depletes ~60% of Tregs for 5 weeks (39), enhanced their LDH3 Ab levels to the level of the immunized-WT female mice. By comparison, male and female WT mice immunized with testis homogenate produce comparable and strong ZAN Ab responses and the male response to ZAN was unaffected by Treg depletion by CD25 Ab. These results indicated that WT male mice are tolerant to the exposed LDH3 and their Tregs maintain the unresponsive state to LDH3. In contrast, male C56BL/6 mice are not tolerant to the sequestered ZAN Ag.

Second, Treg depletion alone (without Ag immunization) resulted in spontaneous EAO and Ab against LDH3 but not against ZAN. Injection of diphtheria toxin depletes 95% of Tregs from the LN and spleen of male B6AF1-DEREG mice that express transgenic diphtheria toxin receptor on Foxp3-positive Tregs (48). After 3 diphtheria toxin injections, 40% of the mice spontaneously develop EAO. The severe testis pathology includes: 1) increase in interstitial accumulation of macrophages with the M1 phenotype, a concomitant reduction in the M2 macrophages and macrophage invasion into the ST; 2) focal peri-tubular immune complexes composed of IgG2 and complement component C3 in 70% of mice; 3) molecular, functional, and ultrastructural evidence of a defective BTB, 4) reduction in spermatogenesis, epididymal sperm content, and testis weight. Because the immunopathology and the LDH3 Ab response in the Treg-depleted DEREG mice are preventable by reconstitution of Tregs from normal WT mice, Treg depletion must be responsible for the LDH3 Ab response and the autoimmune orchitis pathology. Most likely, when Tregs are depleted, the altered innate functional properties of the Ag presenting cells (49) allows the exposed testis Ag, including LDH3, to stimulate pathogenic autoimmune T cell response.

Third, we compared the immune response to OVA between the OVA-Hi mice and OVA-Lo mice that express 40-fold lower level of transgenic OVA in the elongated spermatid (25). The OVA-Hi mice immunized with OVA in adjuvant do not respond to OVA. In contrast, similarly immunized OVA-Lo mice mount a strong Ab and T cell responses to OVA and its T cell epitope and develop severe EAO. Moreover, T cells from their regional LN of the injection site adoptively transfer EAO to OVA-Hi or OVA-Lo recipients but not to the WT BALB/c recipients. Compared with the OVA-Hi mice, only a trace amount of rabbit IgG is detected in <5% of the SFT of OVA-Lo mice injected with rabbit Ab to OVA. The results indicate that although OVA-Lo mice do not expose sufficient OVA to maintain tolerance to OVA, the amount of OVA that egresses from the SFT is sufficient to be presented as target Ag for the effector T cells and Ab to initiate EAO. This finding indicates that the quantity of available target Ag expression also influences Treg-dependent tolerance versus autoimmunity (50). It also indicates that different mechanisms of EAO pathogenesis are responsible for disease induced by high versus low tissue expression or accessibility.

### 2.2 Part II: Unilateral Vasectomy Exposes Epididymal Sperm Ag and Causes Rapid Emergence of ZAN-Specific Tregs and Testis-Specific Tolerance

Because ZAN has the properties of a foreign Ag, we expected it to stimulate a robust Ab response when it becomes exposed. In 2011 and 2013, we tested this hypothesis in WT mice subjected to Uni-Vx, in which the intact contralateral gonad monitors the systemic responses to sperm release in the ipsilateral epididymis. The result from this unintentional study was unexpected.

Epithelial cell apoptosis and desquamation occur in the epididymis within 24 hours after Uni-Vx and sperm are extruded into the interstitial space. To our surprise, the Uni-Vx mice did not have detectable testis Ag-specific or ZAN-specific immune responses for the next 3-4 months (39, 40). This is not because the mice are unresponsive to ZAN. Instead, a response to ZAN emerges only when their Tregs were partially depleted. We confirmed this by two approaches. First, Treg depletion by CD25 monoclonal Ab at the time of Uni-Vx led to a spontaneous Ab to ZAN, CD4 T cell responses to ZAN peptide, and severe bilateral autoimmune orchitis transferable by regional LN CD4 T cells. Second, mice with Uni-Vx alone developed testis Ag-specific tolerance; specifically, the Uni-Vx mice no longer develop EAO when immunized with testis Ag and adjuvants although they developed experimental autoimmune encephalomyelitis when immunized with brain Ag. Importantly, the tolerance state to the testis Ag also depends on Tregs and is preventable by CD25 monoclonal Ab treatment. These findings on ZAN indicate that even sequestered testis Ag, when exposed, can lead to *de novo* testis Ag-specific Treg responses that tempers an excessive immune response against the sequestered self-Ag, as seen in tissue injury and testis infections.

The ZAN-specific Tregs emerge rapidly following uni-Vx. When Treg depletion is delayed for 7 days from the time of Uni-Vx (day 0), the Treg-dependent tolerance against ZAN is no longer reversible (40). The ZAN-specific Tregs most likely represents iTregs since normal mice have no detectable Treg-dependent tolerance to ZAN. In addition, we showed that the Treg induction in uni-Vx is affected in mice with genetic deficiency in PD1 ligand and not in PD1, a finding consistent with the involvement of iTregs in post-transplantation tolerance (51). Interestingly, the Treg-depleted uni-Vx mice produced Ab responses against ZAN but not LDH3, suggesting that sufficient nTregs maintain tolerance to LDH3 despite CD25 monoclonal Ab treatment.

The ZAN-specific tolerance after Uni-Vx is long lasting. Compared to age-matched control mice, the Uni-Vx mice showed resistance to testis Ag induction of sperm Ab and EAO up to 12–16 months (40). However, at 4-5 months after Uni-Vx, low levels of serum ZAN Ab emerge, and the Ab titers amongst individual mice fluctuate over time. We interpret the findings to indicate a gradual decline in Treg control, followed by an imbalance between the Treg and effector T cell responses. The imbalance may be triggered by tissue pathology occurring in the ipsilateral testis and epididymis due to mechanical obstruction of vasectomy. They include severe fibrosis in the epididymis and reduction in spermatogenesis confined to the vasectomized testis (40). In view of these new findings, it is important to further investigate the immune sequelae of clinical vasectomy, a health issue of national and international importance. Moreover, infertility often occurs after vasectomy reversal despite the return of adequate sperm counts (52).

## 3 Summary and Conclusions

**Figure 1:**
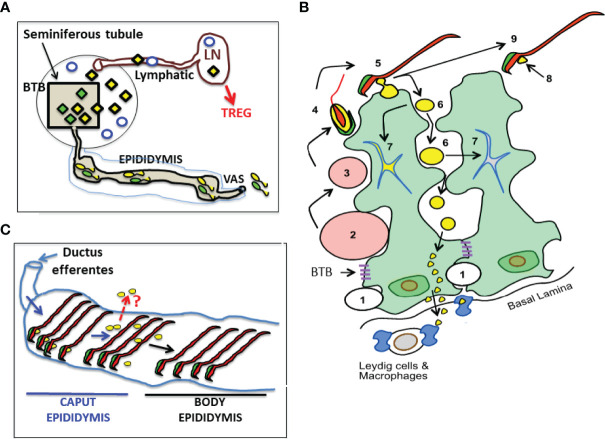
A Landscape of Cellular Antigens in Testicular, Epididymal and Regiona Lymph Node (LN) Compartments. **(A)** The blood-testis barrier (BTB) divides normal testis into the seminiferous tubule and the interstitial compartment. The Ag of the interstitial space, located outside the BTB, including the Leydig cells (empty blue circles), spermatogonia, and preleptotene spermatocytes (not shown) are likely to be exposed. Seminiferous tubule contains all the unique Ag in meiotic germ cells (diamonds). Unexpectedly, some meiotic germ cell antigens (yellow diamonds) continuously egress into interstitial space and reach the regional LN *via* the afferent lymphatic, while other antigens are sequestered (green diamonds). **(B)** A cartoon showing the location and fate of male germ cell development adjacent to two Sertoli cells (with green cytoplasm). Spermatogonia (*1*) traverse the BTB to become spermatocytes (*2 and 3*), and early spermatids (*4*) that develops acrosome (in green), cytoplasm (in yellow), nucleus and tail (in red). Spermiation occurs when late spermatid (*5*) becomes sperm (*9*). Some meiotic germ cell cytoplasm becomes the residual body (RB) (*6*) while others become the cytoplasmic droplet (CD) that attaches to the sperm (*8*). Most RB are engulfed and destroyed by Sertoli cell (*7*). However, some RB stay outside the Sertoli cells until reaching their base, and enter the interstitial space. Systemic Treg-dependent tolerance mechanism protects the meiotic cell antigens in RB and CD (example: LDH3). The sequestered meiotic germ cell antigens (example: ZAN in sperm acrosome) are not protected by systemic tolerance in normal mice. **(C)** The CDs (yellow spheres), initially attached to the sperm in the caput epididymis, soon disappear and are undetectable in the body epididymis. A question (in red): Do the exit of CD Ag in epididymis represent an additional pathway of sperm Ag egress?

We have reviewed the supporting evidence that Ag in the normal testis, relevant to testicular autoimmunity, are not completely sequestered. In **Figure 1**, we illustrate the anatomy of normal mouse testis and the pathways by which Ags in haploid germ cells located behind the BTB can egress from the SFT as contents of the RB. The process appears to involve an undefined exocytosis pathway of the basal Sertoli cells. The exposed testis Ag is presented to Ag-specific Tregs in sufficient quantity to maintain physiological tolerance in normal mice. However, in individuals destined to develop testicular autoimmune disease, the exposed testis Ag can become the immunogen, trigger a pathogenic autoimmune response, and maintain the pathogenic process of EAO (24). EAO can result from a multitude of genetic and environmental factors (53–55), including loss of Treg function, conversion of Treg to effector T cells, response to foreign Ags *via* molecular mimicry (56), and disruption of local regulation (57).

The process of meiotic Ag egress in the SFT also highlights the process of molecular recycling in spermatogenesis that involves the Sertoli cells. In addition to the re-utilization of RB contents in spermatogenesis and spermiogenesis, the apical-to-basal directional movement of macromolecules in the normal SFT epithelium is also documented for the re-utilization of iron in spermatogenesis, and for the recycling of tight junctional molecules in BTB biosynthesis (58–61)

In contrast to the exposed testis Ag, some testicular Ag are normally sequestered behind the BTB. They are normally non-tolerogenic and are not protected by the nTreg. However, when they become exposed, as demonstrated by vasectomy, the normally sequestered Ag can rapidly elicit iTreg that prevent or temper concomitant autoimmune responses against them. Remarkably, the Ag-specific iTreg response is strong enough to maintain tolerance for many months after vasectomy. It is possible that when the sequestered sperm Ag are exposed by bacterial or viral infections, or other types of injuries, they too can induce an iTreg-dependent mechanism for protection (16). Study on the Uni-Vx mice also reveals that major complications of vasectomy result from simple vas obstruction rather than systemic immune mechanism (40).

Our work emphasizes the importance of the Treg-dependent systemic immune protection of male germ cell Ags. It compliments the extensive research on local regulation in the testis, but by no means minimize the importance of local regulation. However, the findings of Treg-depleted DEREG mice indicate that loss of Treg alone can be sufficient to cause EAO. Future research will focus on how the systemic mechanism and local mechanisms are complimentary and function in concert.

## Author Contributions

KT and JH designed the experiments that constitute this review. KT, JH, HQ, KW, CR, AP, DH, YC, and EG conducted the experiments and analyzed the data. JH, DH, EG, YC provided experimental tools or generate the mouse models. KT and JH wrote the manuscript. All authors contributed to the article and approved the submitted version.

## Funding

The study was supported by NIH grants RO1 AI 41236 and RO1 AI 51420.

## Conflict of Interest

The authors declare that the research was conducted in the absence of any commercial or financial relationships that could be construed as a potential conflict of interest.

## Publisher’s Note

All claims expressed in this article are solely those of the authors and do not necessarily represent those of their affiliated organizations, or those of the publisher, the editors and the reviewers. Any product that may be evaluated in this article, or claim that may be made by its manufacturer, is not guaranteed or endorsed by the publisher.
